# LED Wristbands for Cell-Based Crowd Evacuation: An Adaptive Exit-Choice Guidance System Architecture

**DOI:** 10.3390/s20216038

**Published:** 2020-10-23

**Authors:** Miguel A. Lopez-Carmona, Alvaro Paricio-Garcia

**Affiliations:** Departamento de Automatica, Escuela Politecnica Superior, Universidad de Alcala, 28807 Madrid, Spain; alvaro.paricio@coit.es

**Keywords:** crowd evacuation, LED wristbands, behavioral optimization, exit-choice decisions, simulation-optimization modeling, cell-based evacuation

## Abstract

Cell-based crowd evacuation systems provide adaptive or static exit-choice indications that favor a coordinated group dynamic, improving evacuation time and safety. While a great effort has been made to modeling its control logic by assuming an ideal communication and positioning infrastructure, the architectural dimension and the influence of pedestrian positioning uncertainty have been largely overlooked. In our previous research, a cell-based crowd evacuation system (CellEVAC) was proposed that dynamically allocates exit gates to pedestrians in a cell-based pedestrian positioning infrastructure. This system provides optimal exit-choice indications through color-based indications and a control logic module built upon an optimized discrete-choice model. Here, we investigate how location-aware technologies and wearable devices can be used for a realistic deployment of CellEVAC. We consider a simulated real evacuation scenario (Madrid Arena) and propose a system architecture for CellEVAC that includes: a controller node, a radio-controlled light-emitting diode (LED) wristband subsystem, and a cell-node network equipped with active Radio Frequency Identification (RFID) devices. These subsystems coordinate to provide control, display, and positioning capabilities. We quantitatively study the sensitivity of evacuation time and safety to uncertainty in the positioning system. Results showed that CellEVAC was operational within a limited range of positioning uncertainty. Further analyses revealed that reprogramming the control logic module through a simulation optimization process, simulating the positioning system’s expected uncertainty level, improved the CellEVAC performance in scenarios with poor positioning systems.

## 1. Introduction

Uncoordinated crowd behaviors are known for being responsible for pedestrians’ deaths and injuries in crowd evacuations. An efficient evacuation plan is crucial to direct and coordinate evacuees in a safe manner. This coordination can be achieved by deploying guidance systems that provide information for each user on the paths, the exit gates, or the evacuation start time [1].

Many algorithms have been devised for the development of evacuation guidance systems [2]. For example, network flow-based approaches model evacuation planning as a minimum cost network flow problem [3]. The main downside of network flow-based models is that evacuees must follow the paths accurately and fulfill an exact schedule. Various approaches have been suggested to solve this problem using geometric graphs [4]. For example, in [5] a wireless sensor network is partitioned into triangular areas based on the average detected temperature, and safe egress paths are calculated. Following this idea, queuing models [6] build a queuing network to estimate evacuation and congestion delays. Various approaches dynamically develop navigation paths by applying artificial potential fields to the exits and hazards [7,8,9]. This technique suffers from several problems, among which is the convergence time for network stabilization and its search efficiency in scenarios with several exit gates.

There is extensive research on biologically inspired algorithms to search for optimal routes or recommend exits. However, most of this research assumes that a crowd distribution is known in advance, and routes do not adapt to changes during evacuation. For instance, in [10] a multiobjective evacuation route assignment model based on genetic algorithm [11,12] is proposed, while in [13], a shortest path algorithm computes pedestrian routes by iteratively partitioning graph edges at critical division points. Routes are iteratively refined offline until an optimal state is achieved. Among the proposals that adapt to dynamic conditions, the work in [14] proposes bee colony optimization to displace evacuees to safe areas. The downside of this work is the relatively high communication overhead. Another example is a wearable device named LifeBelt proposed in [15] that recommends exits to individuals based on the sensed environment.

As many of existing emergency response systems are built on top of Wireless Sensor Networks (WSN), routing protocols for packet transmission have been adapted to develop guidance systems in emergency scenarios. In [16], an emergency support system built on top of WSN is presented, which is inspired by the cognitive packet network routing [17] in the IoT domain. As communications are essential in an evacuation process, opportunistic communications have also been used in the design of emergency support systems [18].

It is well known that the performance of crowd evacuation processes during emergencies can be strongly affected by exit-choice decision making at the individual level [19,20,21]. Thus, there are research efforts in the area of real-time guidance for crowd evacuations that have focused on studying mechanisms for providing pedestrians with optimal exit-choice information. A promising line of research in this area is that of cell-based evacuation systems [1,22,23]. These systems rely on a cell-based pedestrian positioning infrastructure such that pedestrians in a cell are assumed to receive the same exit gate instructions. In [1], a simulation optimization framework integrates a genetic algorithm and a microscopic pedestrian simulation assignment model. Evacuees are assumed to receive exit-choice indications that may include the optimal start time of evacuation. Similarly, in [24] the idea is to use a gene expression programming to find a heuristic rule. This rule is used to indicate people in the same sub-region to move towards the same exit. The main drawback of these approaches is that they do not consider the dynamic environment features.

As the dynamics of the environment change over time in unpredictable ways, adaptive strategies are recognized as more adequate solutions [21]. There exist adaptive approaches of cell-based evacuation systems, in which the system’s control logic module updates the cells’ exit-choice indications in real-time depending on the existing environmental conditions. In [22], they propose a heuristic rule that considers the distance and width of exit doors as fixed input parameters and density around a given cell as a dynamic parameter. The crowd evacuation planning problem is converted to finding the optimal heuristic rule that minimizes the total evacuation time. To solve this problem, the authors adopt the Cartesian Genetic Programming (CGP) [25]. In [23], we developed an adaptive cell-based crowd evacuation system (CellEVAC) based on behavioral optimization that searches for the optimal evacuation plan through meta-heuristic optimization methodology. As in [22], we obtain adaptive evacuation plans capable of responding to changing environmental conditions. However, our control logic model is easier to configure and optimize, with a more straightforward logic formulation and interpretation, exhibiting a more natural pedestrian behavior.

All these approaches outlined above have mainly focused on the design of algorithms to provide optimal exit-choice indications, assuming that there exist ideal communication and pedestrian positioning infrastructures. However, for a real and effective implementation of this type of system, it is necessary to propose concrete hardware architectures whose deployment is technologically feasible at a reasonable cost. Moreover, given an architectural proposal, it will be essential to evaluate its influence on the performance of the evacuation processes, and if appropriate, propose corrective actions for its improvement.

In this work, we are particularly interested in proposing an adaptive cell-based evacuation system architecture using existing communication and positioning technologies, paying attention to usability, which is essential in emergency evacuations where the information of routing to exit gates should be easily interpretable. Another central question of this study concerns quantifying the influence of pedestrian positioning uncertainty in evacuation time and safety. We would also like to quantify the importance of reprogramming the control logic module under uncertainty conditions by using simulation optimization techniques. Given a control logic optimized assuming an error-free positioning system, what the quantitative benefit of re-optimizing the control logic would be, if we assume a level of uncertainty in pedestrian positioning.

With the purposes mentioned above, this paper proposes a system architecture for our adaptive cell-based evacuation system CellEVAC [23]. The system architecture is based on using light-emitting diode (LED) wristbands to provide exit gate indications to pedestrians. In [23], we assumed an error-free positioning infrastructure, where pedestrians were supposed to be equipped with a generic device (smart-phone or wearable device) with ideal location-aware and color display capabilities. In this paper, the proposed positioning system’s RFID communication channels are modeled using a log-normal propagation model. Finally, we apply the simulation optimization methodology to obtain the control logic subsystem’s optimal configuration under different uncertainty levels. This approach gives us information about the importance of reprogramming the control logic if we know in advance the positioning uncertainty level.

The rest of the paper is organized as follows. Section 2 presents our proposal of system architecture for CellEVAC, the microscopic simulation optimization framework used to perform the experimental evaluation, and the mechanism to model positioning uncertainty. Section 3 presents the experimental evaluation and results. The last section provides concluding comments and possible research extensions.

## 2. Methods

In this section, we first describe the evacuation scenario used in our research. Then, the proposed system architecture and the control logic used to provide optimal indications to pedestrians are presented. To model error in pedestrian positioning, we use a log-normal propagation model introduced in the next subsection. The last two subsections describe the simulation optimization framework and how the simulation optimization experiments were performed, together with the performance metrics used.

### 2.1. Evacuation Scenario

**Figure 1:**
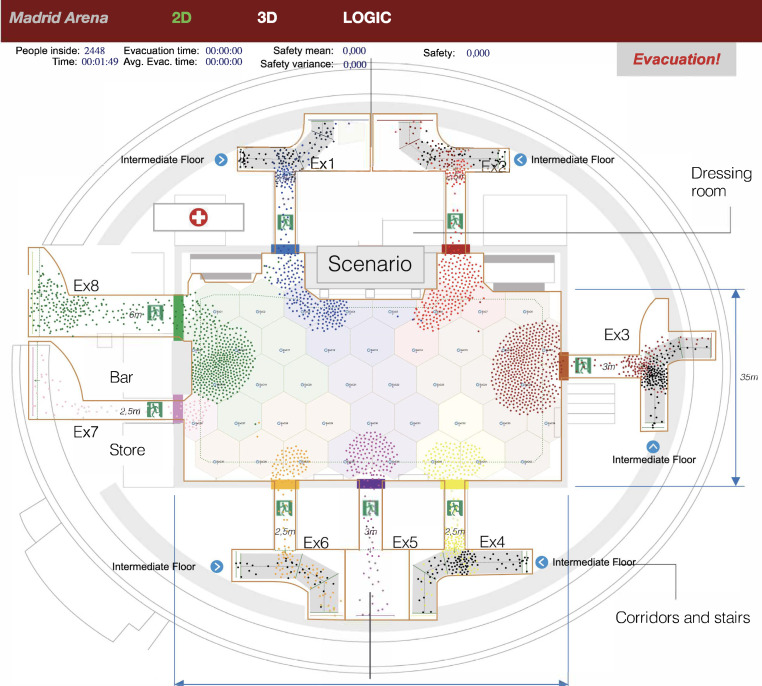
Madrid Arena layout (ground floor). Each colored, thick line represents the color that identifies each exit gate. The colored points define the exit gate selected by each pedestrian.

Our evacuation scenario was Madrid Arena, an indoor arena located in Madrid that hosts sports events, commercial, cultural and leisure activities. It has three floors (access, intermediate, and ground) and 30,000 m^2^, with a capacity of 10,248 spectators. We studied the evacuation of the ground floor, which has a maximum capacity of 3400 spectators with its retractable bleachers removed. Figure 1 shows the ground floor layout, with 1925 m^2^ and eight exit gates (Ex1 to Ex8) with widths in the range 2.5 m and 6 m. Pedestrian flows from intermediate floors were simulated by injecting pedestrians at exits 1, 2, 3, 4, and 6 at the entry points highlighted with a blue dot.

As in [23], we divided the ground floor into 42 regular hexagonal cells of 9 m^2^ and 6 m width, whose dimensions were chosen to provide a balance between control, wireless coverage, and computational efficiency.In this balance, it must be considered that the number of control actions grows exponentially with the number of cells. If the number of control actions increases, obtaining the control logic’s optimal configuration increases in complexity. Moreover, the number of decision changes induced by the system may be significantly higher. On the other hand, if we make the cells very large, the control actions will be less effective because of the lack of granularity. Finally, 9 sqm is a reasonable size for short-range radio transmissions.

### 2.2. System Architecture for CellEVAC

**Figure 2:**
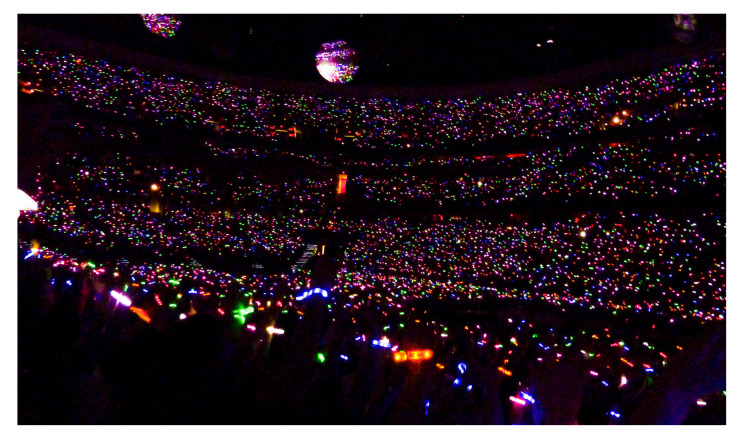
Concert of Coldplay at Verizon Center. Spectators wear LED wristbands manufactured by Xylobands (http://xylobands.com). Author: Matthew Straubmuller; license: https://creativecommons.org/licenses/by/2.0/; source: https://www.flickr.com/photos/imatty35/7550673548.

We considered using radio-controlled LED wristbands that display colors recommending an exit gate. These LED wristbands (Xylobands (http://xyloband.com); CrowdLED (https://crowdled.net)) are widely used at a range of events, from live acts at arenas to product launches, sports matches, parties, and corporate events from 1 to 150,000 people. The wristbands work by creating multiple flash patterns with RGB LEDs that use the full-color spectrum and can be programmed to create visual effects (Figure 2), with typical illuminance values in the range of 70 to 100 lumens. Usually, radio control has a range of hundreds of meters, and the wristbands have a battery life of approximately 20 h. Two extended features that can be found are the inclusion of RFID tags for registration purposes and zonal control to activate wristbands in separate groups.

Our idea was to extend the functionality of these devices, which is oriented towards creating visual effects, using them in case of emergency to guide people to color-illuminated exit gates. The displayed color in the wristband indicates the evacuee the corresponding exit gate. Besides, a synergic effect of using LED wristband lightning is that it may ease image processing for pedestrian flow estimation, which is used in our system to build the control logic.

To make the use of wristbands effective, pedestrians need to know in advance the purpose and functioning of LED wristbands during emergencies. We propose two complementary mechanisms to provide this knowledge. The first mechanism advertises the functionality in the tickets, the online ticket market, and at the facility’s entry points when delivering the LED wristbands to the attendants. Attendants are advised to follow color instructions during an emergency or during the non-emergency evacuation at the end of the event to speed up and favor a comfortable exit from the facility. By extending the functionality of CellEVAC to normal evacuation (the second mechanism), it is expected that the population will progressively get familiar with the system. This approach has another benefit, providing valuable feedback about the system’s performance. Thus, one possible alternative previous to use the system in case of emergencies would limit its use to non-emergency evacuations to perform field studies about its performance.

As described in the introduction section, the proposed system architecture consists of three subsystems:

1. Monitoring and control logic subsystem (Controller Node), which monitors pedestrian flows using image processing and generates exit-choice indications in the form of color allocation to cells.
2. Active RFID cell node network whose purpose is to provide positioning information to pedestrians’ LED wristbands.
3. Radio-controlled LED wristband subsystem, which includes the LED wristbands with color display and radio-frequency (RF) communication capabilities.

**Figure 3:**
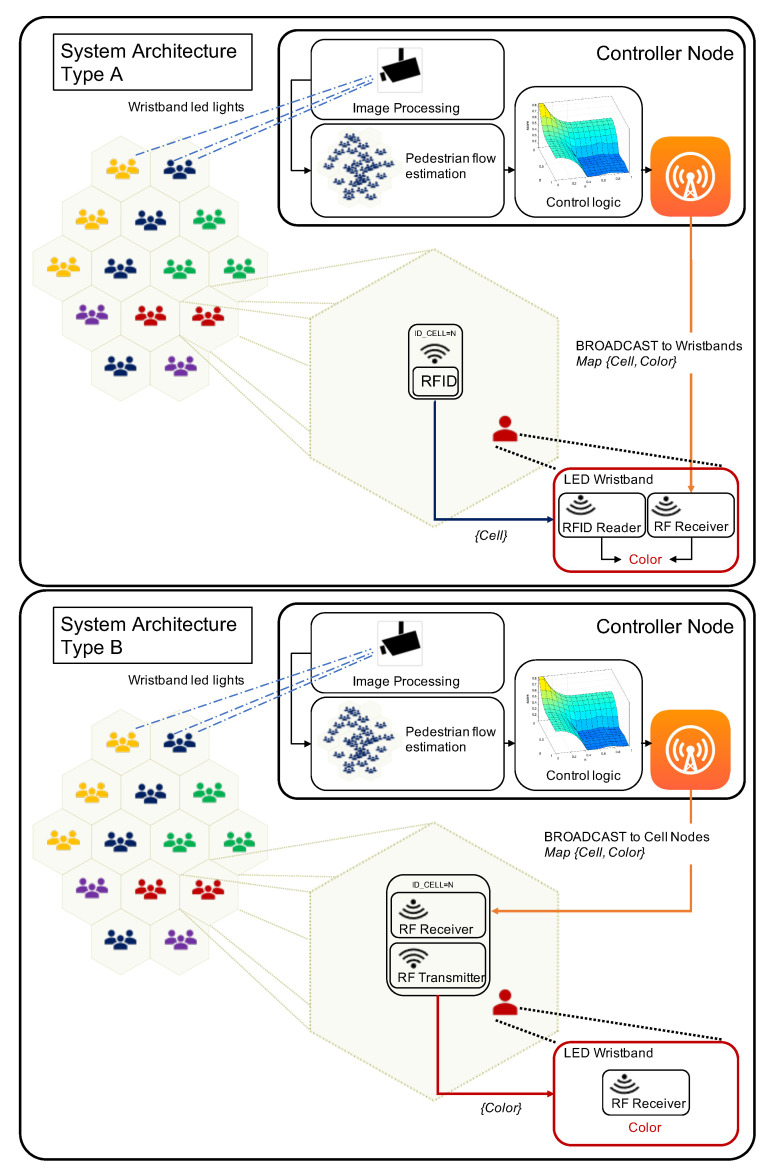
CellEVAC System Architecture: Types A and B.

Figure 3 shows two possible implementations of the system architecture (Types A and B) for deploying CellEVAC using existing off-the-shelf technologies. Both alternatives install a controller node with three functional blocks: pedestrian flow estimation based on image processing, control logic based on behavioral optimization [23], and RF transmitter.

In the controller node, the pedestrian flow estimation block estimates pedestrian density at each cell. This block is assumed to be built on commercially available pedestrian counting technology [26,27,28,29]. Here, two main candidates emerge to be deployed in real implementations of CellEVAC: Time of Flight People (https://www.irisys.net/) counting technology, which is based on signal reflection, or Thermal cameras. Both technologies are not affected by lighting conditions and are effective across wide spaces, providing accuracy levels from 95% to 99% (https://www.trafsys.com/).

Obtained pedestrian densities feed the control logic block that computes the optimal allocation of colors to cell nodes (see Section 2.3), and the RF transmitter broadcasts messages every five seconds containing the 42 tuples {Cell,Color} that assigns a color to each cell. Here, we propose a simple frame format to deliver the messages throughout the RF broadcast communication channel, which can be used in the Type A and B architectures. The frame includes a vector of 42 (cells) * 3 (color bits) = 126 bits, and a header of 16 bits to set the number of cells and color bits. In our scenario, the first byte would be 42 and the second byte 3, stating that each 3 bits sequence corresponds progressively to the color allocated to each cell. The frame ends with trailing bits corresponding to a Cyclic Redundancy Check. Finally, different standard alternatives can be used in the physical layer based on digital modulation such as Frequency-Shift Keying or Phase-Shift Keying. By no means do we mean that this is the only alternative to deliver the messages throughout the broadcast RF channel in the Controller Node, but a realistic and viable alternative.

In the Type A architecture, each cell node is equipped with an active RFID tag [30] that periodically broadcasts its ID (active RFID beacon [31]). On the other hand, the wristbands embed an RFID reader that reads the IDs from the surrounding cells. The wristband selects the ID of the message with the highest Received Signal Strength Indicator (RSSI) to estimate the right pedestrian location [32]. The other element in the wristband is the RF Receiver, which periodically evaluates the broadcast messages with the tuples {Cell,Color} from the controller node. By matching the wristband location (selected cell ID) and cell-color tuples, the wristband lights up with the exit gate color assigned to the cell.

In the Type B architecture, the RF Receiver in the cell node receives the broadcast messages from the controller node with the assigned color. Then, the cell node broadcasts the corresponding color message, which is read by the wristbands. As in the Type A architecture, several broadcast messages from different cell-nodes can be received within a window time. Therefore, the same signal strength selection mechanism is used by the wristbands to select the right color.

The most critical part of this architecture is in the positioning functionality. Both RFID and RF communication channels between the cell node network and wristbands have to cope with a complex signal propagation environment. However, the system does not need to obtain exact position coordinates but select the right cell in which the pedestrians are located. It means that a significant lower location resolution is needed and that there is no need to implement triangulation mechanisms based on RSSI [33]. Another problem to solve is co-channel interference, which may be managed using existing radio resource management schemes [34]. Besides, the RF transmission channel in the controller node is a one-to-many communication channel that has been used to control commercially available LED wristbands in large events for more than a decade, and do not pose a particular challenge.

#### A Procedure to Deliver and Retrieve LED Wristbands

In most existing LED wristband systems (e.g., systems used in large music events), organizers deliver wristbands for free at the facility’s entry points that attendants do not have to return. In the Type B architecture, we may take the same approach because the wristbands embed only an RF receiver and a battery, just as in the existing LED wristbands. Currently, the cost of LED wristbands is around 1$ per unit. Thus, the cost of distributing thousands of wristbands is identical to the cost applicable to current systems and affordable by potential business models based on CellEVAC. It would be necessary to add the flow processing module’s cost and the wired sensor network, which would become part of the building’s infrastructure. This type of equipment is available on the market, and it would only be necessary to embed the code developed in this work to implement the control logic.

Note, however, that in the Type A architecture, the wristbands’ hardware architecture is more complex, adding an RFID Reader and a control logic module used to match cell-position and cell-color. It seems reasonable to assume that the LED wristbands’ cost in the Type A architecture doubles the Type B approach’s cost. There is a trade-off between the LED wristbands’ cost and the sensor network infrastructure cost that we believe favors the Type B architecture.

Independently of the cost of the wristbands and the business model, the green economy drives us to define procedures to reuse the wristbands. The critical point here is to incentivize attendants to return the wristbands at the end of the event. We propose to use the Type B architecture in which the wristbands embed the RF Receiver and a passive tag for identification purposes. This hardware architecture is not new, and it is widely used, as we described at the beginning of the subsection. It is worth mentioning that the cost of integrating a passive tag in the wristbands is only a few cents. Our proposal to incentivize wristbands’ return is to implement a registering process at the facility’s entry points. We assume that attendants purchase tickets online using a personal account. At the entry points, the organization staff registers both the ticket QR code and the wristband RFID tag delivered to the attendant. At the end of the event, the organization reads the RFID tags, paired with their corresponding tickets. Finally, the attendant personal account is updated with confirmation information about the return of the wristband. The incentive could be based, for instance, on future ticket purchase discounts.

### 2.3. Control Logic of CellEVAC and Pedestrian Behavior

The control logic of CellEVAC is based on a behavioral optimization approach proposed in our previous work in [23]. Here, we recall the main concepts that build its operation.

Pedestrians’ exit-choice decision modeling based on discrete choice theory [35] inspired the control logic developed for CellEVAC. Thus, we modeled exit gate color allocation to cell nodes using the simplest and most popular practical discrete choice model, the Multinomial Logit Model (MLM) [35,36]. In the MLM control logic, the controller node allocates exit gates (colors) to cells using a probabilistic model, in which the allocation probabilities of exit gate *j* to cell-node *c* are given by

(1) $$P_{cj}=\frac{\exp(V_{cj})}{\sum_{E_{i}\in E\left(c\right)}\exp\left(V_{ci}\right)}$$

where $E=\{E_{i=1...42}\}$ is the set of exit gates, and $V_{cj}$ is the systematic utility function for cell *c* and exit gate *j*, which is given by

(2) $$\begin{array}{c} V_{\mathrm{cj}}=\beta_{D}\times \frac{DISTANCE_{cj}}{max(DISTANCE)}+\beta_{W}\times \frac{WIDTH_{j}}{max(WIDTH)}\\ \;\;+\beta_{G}\times \frac{GROUP_{cj}-GROUP_{min}}{GROUP_{cj}}+\beta_{E}\times \frac{EXCON_{j}}{criticalDensity_{j}}\\ \;\;+\beta_{C}\left(t\right)\times {NOCHANGING}_{cj} \end{array}$$

The DISTANCE attribute is the distance from cell node *c* to exit gate *j*, while WIDTH represents the width of each exit gate. Both attributes are normalized in the range of 0 to 1.

The third attribute is the GROUP ratio, which estimates the congestion along a path from cell *c* to an exit gate *j*, relative to the least congested path. A group ratio value of 0 indicates that the chosen path is the least congested. When the value of the group ratio tends towards 1, it means that the emptiest path’s imbalance becomes large. The parameter $\beta_{G}$ is expected to be positive if it favors pedestrians to follow other pedestrians and is negative otherwise. Note that with this normalization, we assume that the attribute’s relevance is kept constant throughout the evacuation process.

The fourth attribute EXCON accommodates the congestion at exit gates. For a given density value, congestion is higher if the pedestrian flow is low. We reflect this effect through critical density values obtained from the fundamental diagrams of each exit gate (see in [23]). This $criticalDensity_{j}$ value reflects the density value at which the exit gate’s maximum capacity is reached. Therefore, the $EXCON_{j}$ value representing density at exit gate *j* is normalized by the corresponding $criticalDensity_{j}$ value. This normalization converts EXCON into a unitless attribute around 1. When the value of EXCON is above 1, it means that exit is highly congested. A value close to 0 would indicate that the exit gate is almost empty. In contrast to the normalization procedure used for the GROUP attribute, the distribution of EXCON values exhibits a decreasing evolution as the number of pedestrians in the evacuation scenario decreases. It seems reasonable to assume that the relevance of congestion at exits as a discriminant factor for exit-choice decreases when the overall number of pedestrians is low, and so EXCON is close to 0 at all exits.

The fifth attribute is the NOCHANGING value associated with cell node *c* and exit *j*, which captures how the controller is likely to revise the previous exit gate allocation (this attribute was named PERSONAL in [23]). We treat this attribute as a binary categorical 0–1 value that equals 1 if the current exit gate of cell *c* is *j*, and is 0 otherwise (NOCHANGING=0 ∀k≠j ). Therefore, in a general context, the parameter $\beta_{C}\left(t\right)$ is expected to be positive if the controller tends to maintain the previous exit gate allocations, and is negative otherwise. However, we aimed to modulate the tendency to maintain previous decisions, and so $\beta_{C}\left(t\right)$ is always positive. As was noted above, we assumed that exit-choice changing evolves as evacuation progresses, and therefore the parameter that modulates NOCHANGING is time-dependent. By observing the pattern of behavior under various simulation settings, it was found reasonable that the tendency to maintain decisions increased linearly depending on the current number of pedestrians:

(3) $$\beta_{C}\left(t\right)=\beta_{C}\times \left(1-\frac{numOfPeds(t)}{numOfPeds(t=0)}\right)$$

According to Equation (3), the parameter $\beta_{C}\left(t\to 0\right)$ tends to 0 at the beginning of the evacuation, and so the tendency to revise decisions is higher. As the number of pedestrians decreases, the parameter $\beta_{C}\left(t\right)$ tends to $\beta_{C}$, and the tendency is to maintain previous decisions proportionally to the $\beta_{C}$ value.

In the simulation setting used in this work, we used an update cycle of 5 s. We kept this frequency constant and controlled the frequency of the changes at optimal levels using the parameter $\beta_{C}$.

#### Pedestrian Behavior

In this work, we restrict ourselves to pedestrians that either strictly follow or do not follow the indications provided by CellEVAC during an evacuation. When pedestrians follow the indications, their movement is controlled by the Social Force Model (SFM) [37] that implements the simulator (see Section 2.5), and the exit gate indication provided by CellEVAC through the MLM model described above, whose optimal parameter configuration is shown in Table 1. This configuration under the assumption of perfect pedestrian positioning can be easily obtained through simulation optimization [23].

If pedestrians do not follow the indications, their movement is controlled in the simulator by the SFM, and the selected exit provided by their individual MLM model (different from the model used by CellEVAC). In this case, we use the configuration of parameters of the MLM model defined in [23] that simulates standard pedestrian behavior (see Table 1).

In both scenarios, once a pedestrian selects an exit, pedestrian movement and the path to follow will depend on the destination and the Social Force Model, based on the attractive and repulsive forces existing in the environment at each simulation step. Thus, when using SFM the path that agents follow automatically emerges depending on the environment conditions.

In both models, the group phenomena are captured by the GROUP parameter. However, we do not claim the model is comprehensive, but a reasonable model to capture crowd evacuation movement with parameters currently used in the relevant literature [21,35]. Suppose we wanted to perform a detailed evaluation of hybrid behavior in which a pedestrian may follow or not the indications depending on the environmental conditions in real-time. In that case, the MLM model could be easily extended by adding to the systematic utility function a term $\beta_{S}\times SYS_{j}$ in which $SYS_{j}$ is the indication provided by CellEVAC regarding exit gate *j*. This way, in the exit-choice decisions, pedestrians would consider together the distance, width, group, congestion, decision-changing inertia, and recommendations provided by CellEVAC at the same time.

### 2.4. Modeling Positioning Uncertainty

Active RFID systems are defined by three parts, a reader (wristband), antennas, and a tag (cell node), with their power source. In active RFID applications, RSSI can be used for determining the location of a tag, such that each tag’s RSSI value is proportional to the distance. In our system, the cell-node embeds an active beacon tag that sends out its ID every 3–5 s. Thus, each tag’s RSSI value is proportional to the distance between the wristband and cell node. However, the RSSI value in active RFID applications can be affected by multipath and signal collision [33].

In free space, the relationship of the power transmitted from cell node to wristbands, assuming the antennas are isotropic and have no directivity, is expressed by the free space path loss equation derived from the Friis transmission equation:

(4) $$PL\left(dB\right)=20\log_{10}\left(d\right)+20\log_{10}\left(f\right)-27.55$$

where PL is the free space path loss in dB, *f* is the signal frequency in MHz, and *d* is the distance in meters from the cell node to the wristband. For converting RSSI values into a distance measurement in indoor environments with random shadowing effects, one of the most common approaches taken is to use the log-normal propagation model [38,39]:

(5) $$P_{RX_{dBm}}=RSSI=P_{TX_{dBm}}-PL_{0}-10\eta \log_{10}\frac{d}{d_{0}}+X_{g}$$

where $P_{TX_{dBm}}$ is the transmitted power in dBm, $P_{RX_{dBm}}$ is the received power, $PL_{0}$ is the path loss for a reference distance $d_{0}$ calculated using the free space path loss equation (Equation (4)) or by field measurements, $d\geq d_{0}$ is an arbitrary distance, η is the path loss exponent, and $X_{g}$ is a gaussian random variable with zero mean and variance $\sigma^{2}$ that models the random variation of the RSSI value. The path loss exponent η in indoor environments such as stadiums can reach values in the range of 4 to 7.

User preference or environmental considerations usually prescribe which parameter configuration to use for most applications. In our simulation scenario, we used a frequency of 2.45 GHz, transmission power of 10 W, path loss exponent η=5, and reference distance $d_{0}=1$ m. Thus, RSSI can be expressed as

(6) $$\begin{array}{cc} RSSI & =-60\log_{10}\left(d\right)+X_{g},\;d\geq 1m \end{array}$$

Modifying the variance $\sigma_{g}^{2}$ of the gaussian distribution $X_{g}$ we may modulate positioning uncertainty.

The procedure to calculate each pedestrian’s location in evacuation simulations is a two-step process that repeats every five seconds:

1. Given the set of cell nodes $\left\{c_{i=1...42}\right\}$ obtain the set of distances $\left\{d_{i=1...42}\right\}$ from pedestrian to each cell node $c_{i}$, and calculate the corresponding set {$RSSI_{i}\}$ using Equation (6).
2. If there exists a distance value $d_{i}$ in set $\left\{d_{i=1...42}\right\}$ such that $d_{i}<1$ m, the pedestrian location is $c_{i}$. Otherwise, the pedestrian location corresponds to the cell $c_{i}$ with the maximum $RSSI_{i}$ value.

### 2.5. Microscopic Simulation-Optimization Framework

Much of the related work on crowd evacuations rely on detailed simulations. We opted for a multi-agent microscopic simulation framework based on a Social Force Model (SFM) [37] due to its flexibility and ease of integration of complex interaction and behavior models. Our simulation framework integrates the potential of SFM to mimic physical interactions among evacuees, and of multi-agent systems to simulate complex behaviors and interactions [40].

In this work, the simulation optimization software framework we developed in [23] has been extended with the positioning uncertainty model. The software framework embeds agent-based simulation and discrete event simulation, integrating pedestrian behavior modeling, SFM for pedestrian motion, control logic of exit gate indications, positioning, and optimization features.

We used the commercially available programming, modeling and simulation software packages AnyLogic https://www.anylogic.com/ Accessed 18 August 2020 and Matlab https://www.mathworks.com/ Accessed 19 June 2020. The kernel of the simulation optimization software framework is AnyLogic, which integrates three different modeling methods: discrete event simulation, agent-based simulation, and system dynamics, built on top of a Java-based software development framework. The evacuation scenario layout, pedestrian motion, and evacuation measurements run in AnyLogic, while exit-choice decisions and control logic of exit gate indications are implemented in Matlab. AnyLogic and Matlab are interconnected in a master-slave configuration through the interface with external Java libraries provided by AnyLogic and the Matlab Java API engine (see details below).

**Figure 4:**
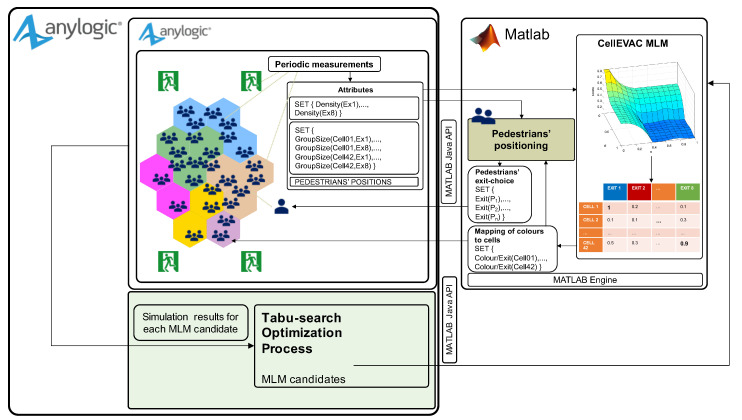
Simulation optimization software framework of CellEVAC with control logic based on Multinomial Logit Model (MLM).

The CellEVAC simulation model with MLM control logic is shown in Figure 4. The evacuation scenario layout, visualization features, and all the functionality regarding the SFM based pedestrian motion were implemented within AnyLogic.

During a simulation, the first step is to send from AnyLogic to Matlab the set of parameters that configure the CellEVAC MLM and Pedestrians’ positioning modules, including the set of cell node center coordinates and exit gates, and the uncertainty level. Next, the pedestrian positioning and densities at exit gates and cells are periodically measured and then transformed into the set of attributes: pedestrian positions, density at each exit gate, and group of pedestrians along the path to each exit. The group size of each pair cell-exit gate is calculated by adding the pedestrians in the cells that are closer to each exit. All these attributes feed the CellEVAC MLM module in Matlab that implements the decision logic to map colors (exit gates) to cells. This mapping is sent back to AnyLogic for visualization purposes, and to the Pedestrians’ positioning module within Matlab to allocate exit gates (colors) to pedestrians (LED wristbands). The Pedestrians’ positioning module implements the function that locates each pedestrian in a given cell-node using the positioning uncertainty model. The output of the Pedestrians’ positioning module is the set of pairs pedestrian-exit gate, which is sent to AnyLogic for simulating pedestrian motion.

To search for optimal configurations of the MLM model, we used a simulation optimization process that adopts a Tabu-Search algorithm (TS) [41], which iteratively searches the solution space. At the same time, the microscopic crowd simulation guides the search by evaluating the evacuation time and safety of the solutions generated by the TS algorithm. The optimization process is built on top of the OptQuest https://www.opttek.com/ Accessed 18 August 2020 optimization engine provided by AnyLogic. Figure 4 shows the optimization module on a green background. The parameters of the CellEVAC MLM model are the “MLM candidates” generated by the TS algorithm. Thus, each candidate is defined by a tentative set of parameters β sent to the MATLAB Engine at each iteration of the optimization process. The simulations results are sent back to the optimization module for its evaluation and thus guide the optimization process.

### 2.6. Simulation-Optimization Experiments

The performance measurements in all the experiments were the total evacuation time, average safety, variance of safety, and the average number of pedestrians’ exit-choice decision changes. The average and variance of safety are based on the safety values computed at the different exit gates. The procedure to calculate exits’ safety values is first to obtain their Fundamental Diagrams (FD) derived through microscopic simulation. This process is described in detail in [23]. Below, we summarize the process to improve readability.

**Figure 5:**
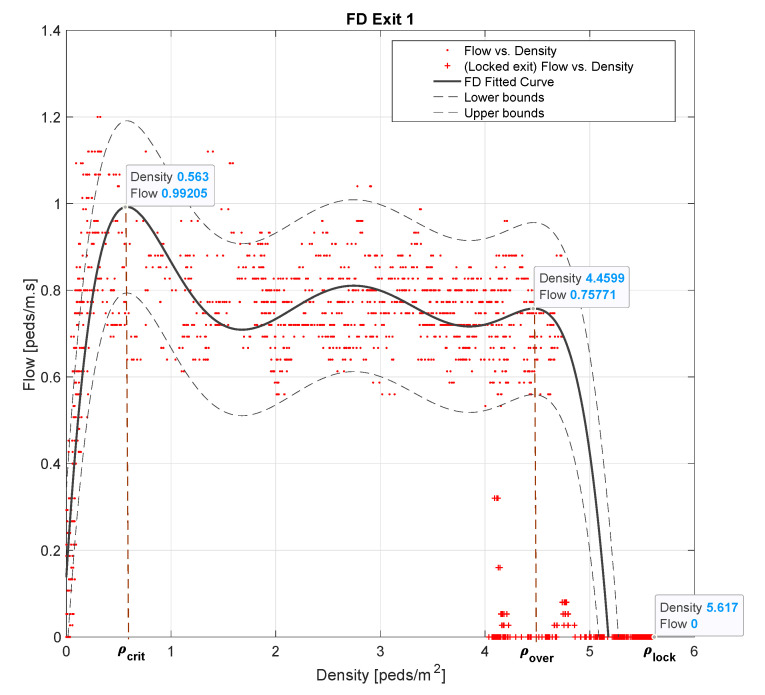
Fundamental diagram of exit 1.

A FD represents the relation between pedestrians’ flow and density. To obtain each FD, we simulate pedestrian flows heading to each exit. Each flow is linearly increased for 10 min to exceeding capacity and then linearly decreased. This sequence is repeated to simulate queue build-up and recuperation phases until a simulation interval of one hour is completed. At minute 50, the exit is locked to characterize pedestrian dynamics in the event of a fall. Density is measured in an area defined by the four closest cells to each exit while we measure pedestrian flow at each exit gate. Figure 5 illustrates the Fundamental Diagram obtained for Exit 1. We refer to the work in [23] that shows the FDs for all the exits.

In red points are represented the flow vs. density measurements, while the red crosses show the blocking phase. Using curve fitting, we characterize the different possible phases of pedestrian flow. The critical density $\rho_{crit}$ delimits the free-flow region. As pedestrian flow increases, a fast backpropagation shockwave is formed that carries the density value to $\rho_{over}$. This stable state maintains as long as the arc formation due to high pressure is present. During the locking phase, the density value increases from $\rho_{over}$ to $\rho_{lock}$ due to queue accumulation.

These density thresholds are used to build the safety metric at each exit gate *j*:

(7) $$Sf_{j}=\left(-\bar{\rho_{j}}-\gamma \cdot \sigma_{j}^{2}\right)\times 100,$$

such that

(8) $$\begin{array}{cc} \bar{\rho_{j}} & =\frac{1}{N}\sum_{n=1}^{N}\frac{\rho_{j}^{\prime }\left(n\right)-\rho_{sf_{j}}}{\rho_{lock_{i}}-\rho_{sf_{j}}}\;, \end{array}$$

(9) $$\begin{array}{cc} \sigma_{j}^{2} & =\frac{1}{N}\sum_{n=1}^{N}{\left(\frac{\rho^{\prime }\left(n\right)-\rho_{sf_{j}}}{\rho_{lock_{j}}-\rho_{sf_{j}}}-\bar{\rho_{j}}\right)}^{2}\;, \end{array}$$

with

(10) $$\begin{array}{cc} \rho_{j}^{\prime }\left(n\right) & =\left\{\begin{array}{cc} \rho_{j}\left(n\right) & \rho_{j}\left(n\right)\geq \rho_{sf_{j}}\\ \rho_{sf_{j}} & \rho_{j}\left(n\right)<\rho_{sf_{j}} \end{array}\right.\;, \end{array}$$

(11) $$\begin{array}{cc} \rho_{sf_{j}} & =f(\rho_{crit_{j}},\rho_{over_{j}},\rho_{lock_{j}}) \end{array}$$

Given a set of *N* density values measured at exit gate *j*, Equations (8) and (9) give the time-mean density and density time-variation values in Equation (7), respectively, which are weighted by γ. Density time-variation measures the negative impact of variations of pedestrian flow. Both terms are normalized to 1 using the range defined by $\rho_{lock_{j}}$ and a predefined threshold $\rho_{sf_{j}}$. According to the equations, if exit gate *j* is locked, $Sf_{j}=-100$, while in a safe scenario where densities are always below $\rho_{sf_{j}}$, $Sf_{j}$ equals 0.

The value given to $\rho_{sf_{j}}$ is defined as a function of $\rho_{crit_{j}},\rho_{over_{j}}$ and $\rho_{lock_{j}}$. Since in our study $Sf_{j}$ is used primarily for comparison purposes, as in [23], f(.) is defined merely as the following weighted-average function,

$$\rho_{sf_{j}}=0.9*\rho_{crit_{j}}+0.1*\rho_{over_{j}}$$

We make γ equal to 5 to strengthen the influence of density time-variations. Finally, the average and variance of the safety values at the exit gates are obtained as follows.

$$\begin{array}{cc} Sf= & \frac{1}{\left|E\right|}\sum_{j=1}^{\left|E\right|}Sf_{j}\;,\\ Sf_{var}= & \frac{1}{\left|E\right|}\sum_{j=1}^{\left|E\right|}{\left(Sf_{j}-Sf\right)}^{2} \end{array}$$

The variance of safety $Sf_{var}$ is used to estimate the imbalance of safety between the exit gates.

Using the performance measurements described above, we conducted two types of experiments: (i) sensitivity analysis to positioning uncertainty and (ii) simulation optimization. In all the simulation set-ups, the evacuee population consisted of 3400 pedestrians on the ground floor, who had a preferred evacuation speed obtained from a uniform distribution between 1.24 and 1.48 m/s. To speed up the simulation optimization experiments, we imposed a deadline of 25 min to each evacuation simulation iteration, after which the simulation iteration was aborted.

Two different evacuation scenarios were considered depending on the experiment: evacuations without external flows (NEF) in which no pedestrians were coming from the upper floors, and evacuations with external flows (EF) (i.e., with pedestrians coming from the upper floors) to simulate more complex pedestrian flow interactions. In EF scenarios, three exit gates were chosen at random at each simulation iteration. Two of these exit gates received an incoming pedestrian flow rate of 120 peds/m, while the third exit gate was blocked.

In the sensitivity analysis experiments, each experiment ran the evacuation simulation model multiple times varying the positioning uncertainty level (standard deviation $\sigma_{g}$ of the Gaussian distribution $X_{g}$) from 0 dB to 40 dB at discrete steps, showing how the simulation output (i.e., the performance measurements) depended on it. To evaluate up to which uncertainty level is beneficial CellEVAC in comparison with not using a guidance system, we also included the case in which pedestrians did not use the CellEVAC system.

Due to the stochastic nature of the evacuation processes, we used a replication algorithm to obtain representative results for a given parameter setting and a specific simulation output. This algorithm defines a minimum and a maximum number of experimental runs per parameter setting (replications of a simulation), a confidence level for the sample mean of replications (simulation output average), and an error percent. The minimum number guarantees the minimum number of replications, while the confidence level and error percent determine if more replications are needed. Simulations for a given parameter configuration stops when the maximum number of replications has been run or when the confidence level is within the given percentage of the mean of the replications to date. In our experimental set-up, evacuation time was used as an output parameter to control the number of replications between 10 and 50. The confidence level was fixed to 95%, and the error percent to 0.5.

In the simulation optimization experiments, we used the Tabu-search optimization algorithm [41]. The goal was to find the optimal combination of parameters of the MLM model that resulted in the best possible solution. We considered two different optimization scenarios characterized by the fitness function (objective function) used and the existence of external pedestrian flows.

- NEF: Optimize Time and Safety (min(evacTime−Sf)) without External Flows. The goal is to optimize evacuation time and average safety, and the evacuation scenario does not include external pedestrian flows.
- EF: Optimize Time and Safety (min(evacTime−Sf)) with External Flows. The goal is to optimize evacuation time and average safety, and the evacuation scenario includes external pedestrian flows.

As in the sensitivity analysis experiments, the optimization algorithm applies a replication algorithm. However, while in the sensitivity analysis, the number of replicas was limited by the evacuation time value, in simulation optimizations, the stop condition was controlled by the fitness function (objective function).

## 3. Results

### 3.1. Sensitivity Analysis of Positioning Uncertainty

**Figure 6:**
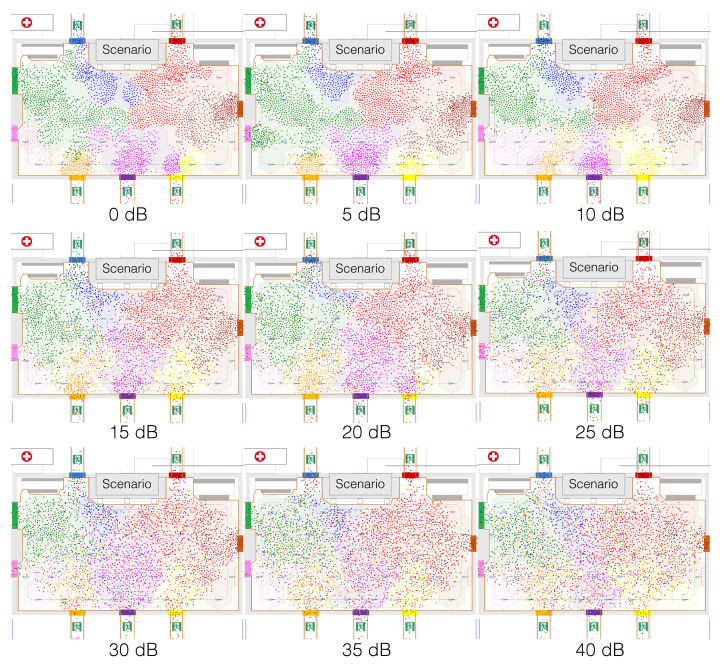
Still images 25 s after the start of the evacuation from single run simulation experiments for different standard deviation values $\sigma_{g}$. The cells are shaded with the exit-gate color allocated by the controller node. Colored dots represent pedestrians with the colors shown by their LED wristbands.

For illustration purposes, Figure 6 shows still images 25 s after the start of the evacuation for different standard deviation values $\sigma_{g}$, from 0 dB to 40 dB. As expected, the snapshots exhibited a progressive level of error in cell detection, becoming more evident from 15 dB.

**Figure 7:**
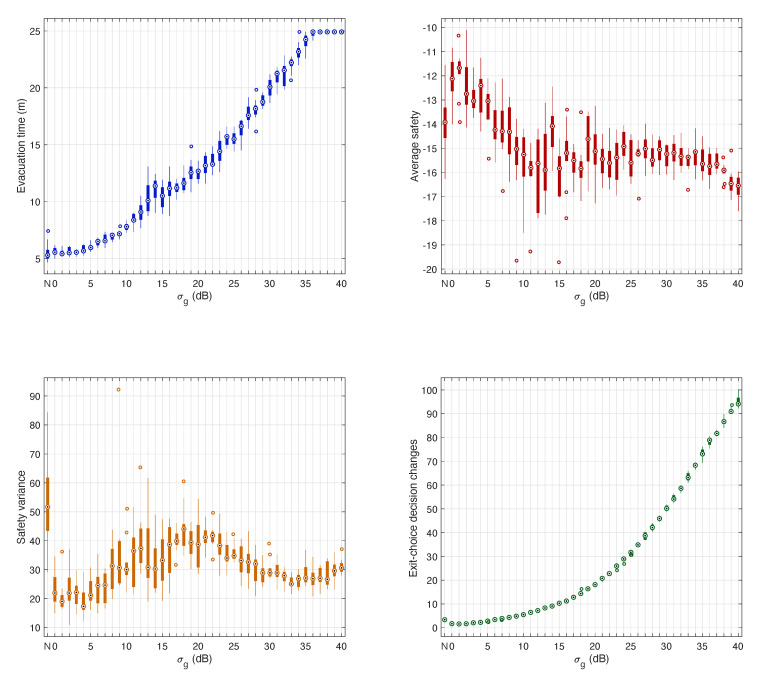
Sensitivity analysis of the positioning uncertainty in evacuation scenarios without external pedestrian flows. The box-plots on the first row show the sensitivity of evacuation time and average safety to standard deviation values $\sigma_{g}$ in the range 0 dB to 40 dB. The second-row plots show the sensitivity of safety variance and the number of decision changes to the standard deviation values $\sigma_{g}$. In the four box-plots, $\sigma_{g}=N$ represents an evacuation scenario in which pedestrians do not use CellEVAC.

In evacuation scenarios without external flows (Figure 7), results revealed that evacuation time increased linearly for $\sigma_{g}$ above 5 dB. Regarding safety, increasing values of $\sigma_{g}$ had a significant negative impact on average safety, though for $\sigma_{g}$ above 10 dB average safety stabilized around −15. Besides, the impact on safety variance was not so significant as in average safety. As expected, performance worsened for increasing $\sigma_{g}$, though for values above 20 dB safety variance tended to improve and stabilize. At the cost of an increasing evacuation time, we observed how uncoordinated pedestrians’ movement when positioning uncertainty was high, made spatial-density at exit gates decrease, and so safety measurements stabilize or improve. The number of exit-choice decision changes increased exponentially with $\sigma_{g}$, due to the logarithmic scale (dB) used to define the values of $\sigma_{g}$.

When compared to evacuations without external flows in which CellEVAC did not operate, and not considering the safety variance, we observed that using CellEVAC was beneficial strictly for values of $\sigma_{g}$ below 5 dB. Higher values of $\sigma_{g}$ could be valid at the cost of an increase in evacuation time. However, note that not using CellEVAC comes at the cost of a significantly higher safety variance.

**Figure 8:**
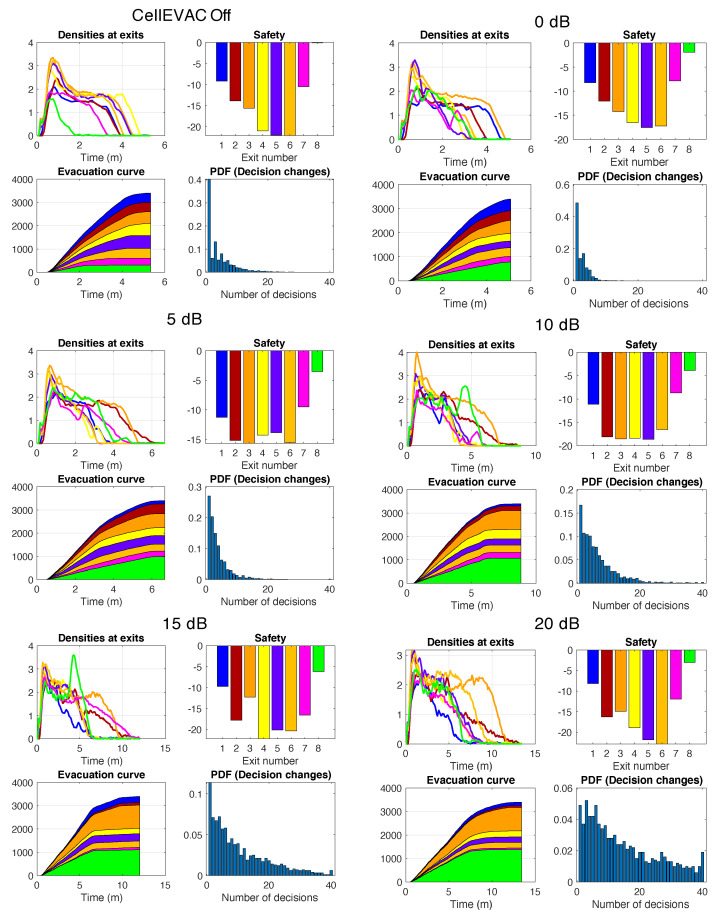
Results of single run simulations without external flows in evacuation scenarios in which pedestrians do not use CellEVAC (CellEVAC Off), and in evacuation scenarios using CellEVAC and assuming positioning errors from 0 to 20 dB. Each subfigure shows the evolution of density, safety values and evacuation curves at each exit gate, and the histogram (probability density function) of the number of decision changes of the pedestrians. Colors identify the different exit gates, as described in the Madrid Arena layout.

Figure 8 presents the outcomes of single run simulations in scenarios without external flows. As expected, when CellEVAC is not used, pedestrian flows are unbalanced. Exit 8, which is the exit with the highest capacity, is underutilized. The system performs reasonably well up to 10 dB, with a more balanced behavior. However, as error positioning increases, the number of decision changes becomes unmanageable, and evacuation time goes to 13 min for 20 dB, which is not acceptable.

**Figure 9:**
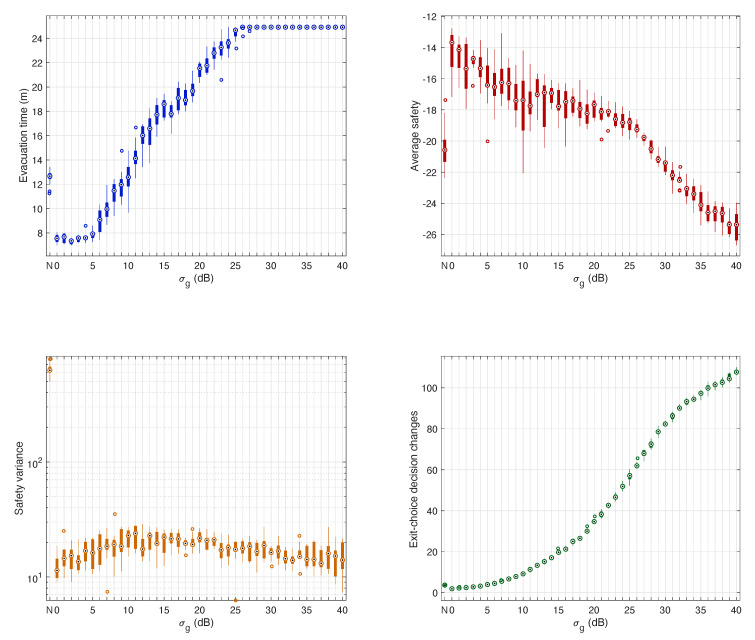
Sensitivity analysis of the positioning uncertainty in evacuation scenarios with external pedestrian flows. The box-plots on the first row show the sensitivity of evacuation time and average safety to standard deviation values $\sigma_{g}$ in the range 0 dB to 40 dB. The second-row plots show the sensitivity of safety variance and the number of decision changes to the standard deviation values $\sigma_{g}$. In the four box-plots, $\sigma_{g}=N$ represents an evacuation scenario in which pedestrians do not use CellEVAC.

In evacuation scenarios with external flows, the sensitivity analysis results revealed the same trend as without external pedestrian flows (Figure 9). When compared to evacuations that did not use CellEVAC, and not considering the safety variance, the benefit of CellEVAC expanded to $\sigma_{g}$ below 10 dB. However, note that safety variance is exceptionally high when not using CellEVAC, which means that pedestrian flows at different exit gates is highly unbalanced.

**Figure 10:**
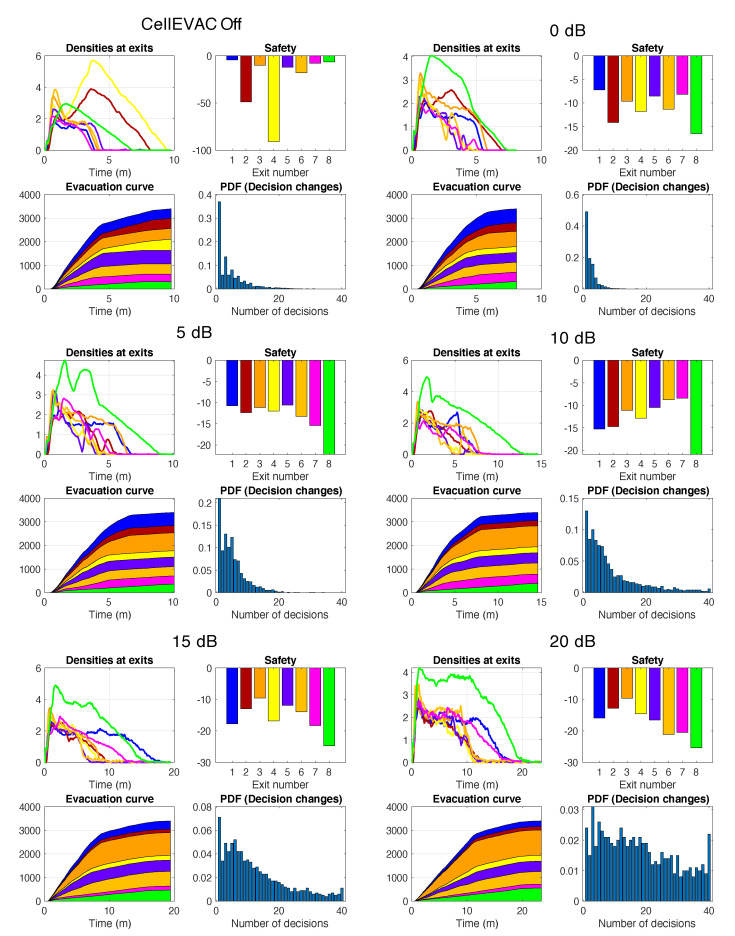
Results of single run simulations with external flows in evacuation scenarios in which pedestrians do not use CellEVAC (CellEVAC Off), and in evacuation scenarios using CellEVAC and assuming positioning errors from 0 to 20 dB. Each subfigure shows the evolution of density, safety values and evacuation curves at each exit gate, and the histogram (probability density function) of the number of decision changes of the pedestrians. Colors identify the different exit gates, as described in the Madrid Arena layout.

Figure 10 presents the outcomes of single run simulations in scenarios with external flows. External flows in all the simulations were injected at exits 2 and 4, while the entry at exit gate 8 was blocked. Pedestrians’ speed was artificially reduced by 100 in a restricted area at the entrance of the exit gate 8 to simulate that an external event blocked the exit. We may observe the same trends as in the single run simulation without external flows. As an example, when CellEVAC is off, Exit 4 (yellow) gets a safety value close to −100, while with CellEVAC safety values are homogeneous and around −10, and flows adapt to the dynamic conditions of the environment.

Overall, the results of the sensitivity analyses for scenarios with and without external flows suggest a clear benefit of using CellEVAC if the positioning system exhibits RSSI random variations below 10 dB.

### 3.2. Optimizing CellEVAC for Different Positioning Uncertainty Levels

**Figure 11:**
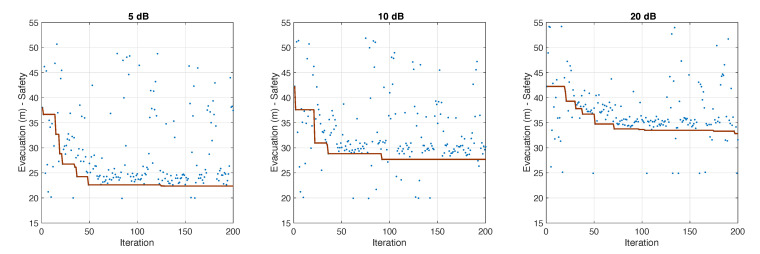
Progress of the Tabu-search simulation optimization of the MLM models that implement the CellEVAC guidance system for $\sigma_{g}$ equal to 5, 15 and 20 dB. Solutions below the current best solution (red line) correspond to non-viable solutions.

Figure 11 shows the progress of the Tabu-search simulation optimization of the MLM models’ parameter configurations for values of $\sigma_{g}$ equal to 5, 15 and 20 dB. The objective was to optimize evacuation time and average safety in scenarios with external flows. It was assumed that the entire population of evacuees followed the indications of the CellEVAC system. Furthermore, we imposed an arbitrary simulation stop-limit of 25 min to evacuation time, and a restriction to the viability of the solutions was incorporated to remove solutions in which there were pedestrians pending evacuation.

The optimal parameter configurations found are shown in Table 2. We did not observe significant differences between the different parameter configurations, except for the $\beta_{D}$ and $\beta_{G}$ parameters. The distance parameter gained more influence in scenarios with high positioning uncertainty. Remarkably, for $\sigma_{g}=20$ dB, the group parameter $\beta_{G}$ had a positive sign. Our interpretation is that a higher influence of distance and a positive value of $\beta_{G}$ contributes to more uniformity in the exit gate indications and less scattering in color allocation to cells. As a consequence of this, the probability of location error decreases.

**Figure 12:**
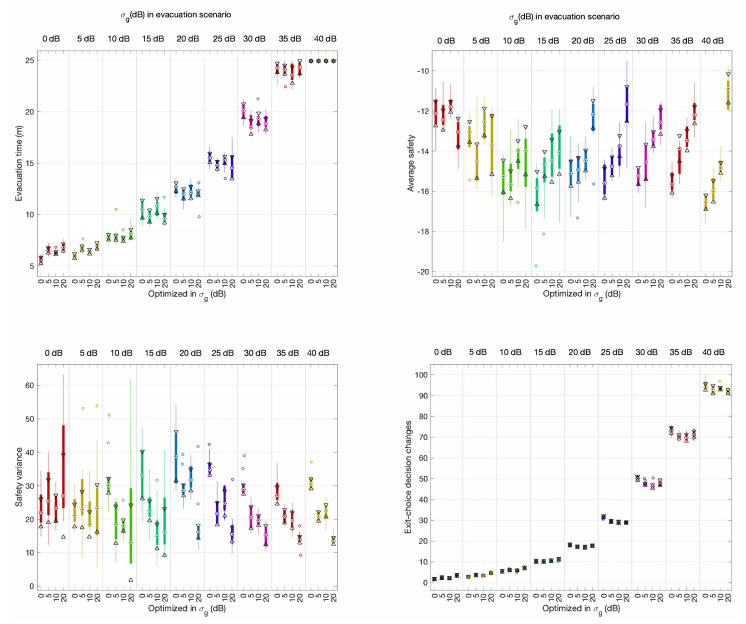
Median box-plots of the performance measurements of the optimal configurations of CellEVAC for $\sigma_{g}=0,5,10,20$ dB, in evacuation scenarios without external pedestrian flows and with positioning uncertainty levels from 0 dB to 40 dB in steps of 5 dB. Bottom horizontal axes categorize the optimal configuration of parameters used (0, 5, 10, or 20 dB) to configure CellEVAC. Upper horizontal axes categorize the $\sigma_{g}$ value that models the positioning system in the evacuation scenario. For instance, a value of 25 dB in the axis “$\sigma_{g}$ (dB) in evacuation scenario” and 15 dB in “optimized in $\sigma_{g}$ (dB)” expresses that CellEVAC has been configured to use the optimal configuration found with 15 dB, and that it is tested in an evacuation scenario with $\sigma_{g}=25$ dB. The triangles in the box-plots display the variability of the median between samples. If the notches of two measurements do not overlap, they have different medians at the 5% significance level.

**Figure 13:**
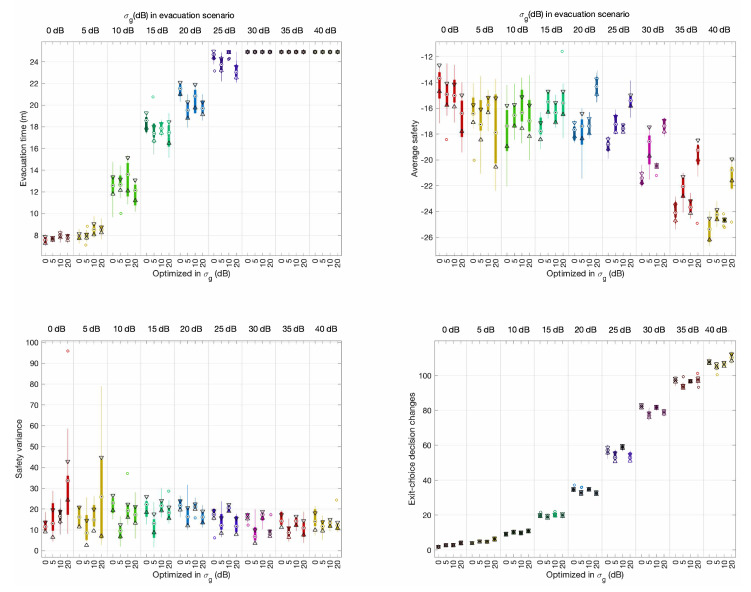
Median box-plots of the performance measurements of the optimal configurations of CellEVAC for $\sigma_{g}=0,5,10,20$ dB, in evacuation scenarios with external pedestrian flows and with positioning uncertainty levels from 0 dB to 40 dB in steps of 5 dB. Bottom horizontal axes categorize the optimal configuration of parameters used (0, 5, 10, or 20 dB) to configure CellEVAC. Upper horizontal axes categorize the $\sigma_{g}$ value that models the positioning system in the evacuation scenario.

The optimal configurations found were tested in evacuations with different positioning uncertainty levels, from 0 dB to 40 dB in steps of 5 dB. The results have been summarized in Figure 12 and Figure 13 under evacuation scenarios without and with external flows, respectively.

In evacuation scenarios without external flows (Figure 12), average evacuation time, and exit-choice decision change performance measurements did not show significant differences between the different configurations and evacuation scenarios. Interestingly, we found a positive trend in the results in terms of average safety and safety variance for evacuation scenarios for 20 dB and above when using the optimal configuration found for 20 dB. In evacuation scenarios with external flows (Figure 13), the results were similar except for safety variance, for which we did not observe any improvement.

The results presented in Section 3.1 show that CellEVAC is useful only if the positioning system exhibits RSSI random variations below 10 dB. Besides, the performance analysis results of the optimal configurations do not exhibit any improvement below 20 dB. Consequently, we can conclude that there is no evidence that optimizing the MLM model under the assumption of an expected random variance of RSSI contributes to an improvement in the performance of the CellEVAC system.

## 4. Conclusions

Our use of an MLM model to implement the control logic of cell-based crowd evacuation systems has proven to be very efficient (see [23]). However, as in other existing works on cell-based crowd evacuation systems [1,13,24], little attention has been paid to propose a system architecture based on existing technologies and assuming real communication and positioning infrastructures. In our opinion, these considerations are crucial to boost the real implementation of these systems.

In this paper, we have proposed a specific system architecture built upon radio-controlled LED wristbands that connect with a cell node network and a controller node through radio-frequency communication channels. The use of LED wristbands has numerous advantages, among which we highlight its low cost, being a technology widely used to create visual effects at large events, and being an intuitive and straightforward interface that can make it exceptionally efficient in emergencies. This type of indication system greatly simplifies the interpretation of exit gate indications, which is particularly important in stressful situations found typically in evacuation scenarios. Indirectly, it can also improve the accuracy of the detection of pedestrian flows in the controller node.

Another of our aims was to quantitatively study the sensitivity of evacuation time and safety to uncertainty in the positioning system. With this objective, we have modeled the communication channel between the LED wristbands and the cell nodes using a log-normal propagation model. To model different levels of uncertainty in positioning, we have modulated the random variations of RSSI from the propagation model. In the sensitivity analysis of performance parameters to different values of RSSI variance, CellEVAC is shown to be operational strictly up to values of 10dB. The system generates too many color changes in the wristbands and a significant increase in evacuation times for higher values.

Our last goal was to evaluate if it was possible to improve the CellEVAC performance obtaining new optimal MLM parameter configurations in which different levels of RSSI standard deviation were assumed. The results obtained have revealed that improvements found are relevant only for evacuation scenarios with levels of positioning uncertainty greater than 20 dB, in which CellEVAC is not operational. Thus, to optimize the MLM model used in the CellEVAC control logic, it is valid to assume that the positioning system is error-free. However, the system cannot be applied in a real environment if the standard deviation of the RSSI values is greater than 10 dB.

Several extensions are being considered for this research, mainly focused on investigating how to expand the allowed range of RSSI variation without the need to modify the existing positioning infrastructure. Another research extension is related to developing a prototype of the CellEVAC system architecture proposed in this paper. We are aware that the behavioral issues in this type of system are critical, mainly in emergencies, and so a validation of the proposed mechanisms is needed. As we proposed in Section 2, a possible validation of the system would be based on activating the system during the standard/regular evacuation process (not in an emergency). We believe that this strategy can be easily replicated over time in the same facility to obtain feedback about the penetration rate and the usefulness in terms of evacuation time and safety. This strategy would also instruct people on how LED wristbands work and generalize this functionality’s knowledge. Moreover, a small-scale experiment can be designed to calibrate and validate the propagation model previous to this validation strategy. This would serve to evidence which specific hardware infrastructure would be needed that can guarantee a certain performance level. 

## Figures and Tables

**Table 1:** Optimal parameter configuration for the CellEVAC MLM decision logic model, and parameter configuration of the MLM pedestrians’ standard behavior model.

	$\beta_{D}$	$\beta_{G}$	$\beta_{E}$	$\beta_{W}$	$\beta_{C}$
Optimal CellEVAC for 0 dB	−17.723	−2.181	−1.671	1.064	2.594
Standard (without CellEVAC)	−28	0.6	−0.5	0.6	0

**Table 2:** Optimal parameter configurations of the MLM model for different values of $\sigma_{g}$.

$\sigma_{g}$	$\beta_{D}$	$\beta_{G}$	$\beta_{E}$	$\beta_{W}$	$\beta_{C}$
0 dB	−17.723	−2.181	−1.671	1.064	2.594
5 dB	−16.040	−3.224	−2.267	0	6.816
10 dB	−17.696	−2	−2	1.685	3
20 dB	−28.479	10	−3.083	0.041	4.025
